# A pro-BMP function exerted by *Rhodnius prolixus* short gastrulation reveals great diversity in the role of BMP modulators during embryonic patterning

**DOI:** 10.1098/rsob.230023

**Published:** 2023-07-05

**Authors:** M. Berni, J. Mota, D. Bressan, L. Ribeiro, G. Martins, J. Pereira, I. Ramos, R. Nunes-da-Fonseca, H. Araujo

**Affiliations:** ^1^ Institute for Biomedical Sciences, Federal University of Rio de Janeiro, Rio de Janeiro, Brazil; ^2^ Institute of Biodiversity and Sustainability (NUPEM), Federal University of Rio de Janeiro, Campus Macaé, Rio de Janeiro; ^3^ Institute for Medical Biochemistry, Federal University of Rio de Janeiro; ^4^ Instituto Nacional de Ciência e Tecnologia em Entomologia Molecular, Brazil (INCT-EM); ^5^ Post-graduate Program in Morphological Sciences (PCM), Federal University of Rio de Janeiro

**Keywords:** bone morphogenetic proteins, dorsal–ventral patterning, BMP modulators, evolution, *Rhodnius prolixus*, chagas disease

## Abstract

Dorsal–ventral (DV) patterning is regulated by the bone morphogenetic pathway (BMP) in Bilateria. In insect DV patterning, the Toll pathway also plays a role, in addition to BMPs. Variations in the relative importance of each pathway for DV patterning have been reported using single species of coleopteran, hymenopteran, hemipteran and orthopteran insects. To investigate if the molecular control of DV patterning is conserved inside an insect order, the emergent model hemiptera species *Rhodnius prolixus* was studied. We found that *R. prolixus* BMP pathway controls the entire DV axis, with a broader effect respective to Toll, as shown for the hemiptera *Oncopeltus fasciatus.* Different from *O. fasciatus*, the unique *R. prolixus short gastrulation* (*sog*) and the *twisted gastrulation* (*tsg*) orthologues do not antagonize, but rather favour embryonic BMP signalling. Our results reinforce the hypothesis that hemiptera rely preferentially on BMPs for DV patterning but that, surprisingly, in *R. prolixus* Sog and Tsg proteins exert only a positive role to establish a dorsal-to-ventral BMP gradient. Since *sog* has been reported to be lost from orthopteran and hymenopteran genomes, our results indicate that Sog's role to modify BMP activity varies greatly in different insect species.

## Graphical abstract

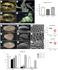

## 1. Introduction

A conserved feature of Bilateria is the establishment of embryonic dorsal–ventral (DV) gene expression territories by the action of bone morphogenetic proteins (BMPs; reviewed in [1]). BMPs act as morphogen gradients, their graded distribution along the tissue defined by extracellular modulators. In the frog *Xenopus laevis*, the Spemann organizer secretes the BMP antagonists noggin, follistatin and chordin, and the embryo lacks dorsal structures if all three are knocked down with morpholino oligonucleotides [2]. Expressed in animals that display an inverted DV axis relative to each other, vertebrate chordin and the *Drosophila* homologue, Short gastrulation (Sog), ensure that the neural tissue is produced in the dorsal and ventral regions of the ectoderm, respectively (reviewed in [1]). Heterologous expression assays support a homologous and ancestral function for BMP pathway elements in the establishment of the deuterostome and protostome DV axis [3].

In some insect groups, loss-of-function for the *BMP2/4* homologue *decapentaplegic* (*dpp*) results in the restricted loss of dorsal elements, such as the extraembryonic tissues in the diptera *Drosophila melanogaster* [4] and *Musca domestica* [5], as well as in the beetle *Tribolium castaneum* [6] and the cricket *Gryllus bimaculatus* [7]. In other species, however, loss of BMP function results in broad alterations in patterning, as revealed with knockdown assays for BMP encoding genes in the wasp *Nasonia vitripennis* [8] and the bug *Oncopeltus fasciatus* [9], where all DV patterning is lost. The differences in the contribution of the BMP pathway to insect DV patterning are paralleled by an inverse contribution of the Toll pathway to axial patterning. For instance, Toll signals are responsible for setting the entire DV axis in *Drosophila* [10] and *Tribolium* [11], while they exert only a ventral polarizing role in *O. fasciatus* [9]. It is currently discussed whether the differences in the respective contribution of the Toll and BMP pathways to DV patterning in the insect clade are the result of independent gains of broad Toll function by convergent evolution or based on the recurrent restriction of Toll function along different orders [7]. Examining the genetic basis of DV patterning in additional species should help define the most parsimonious sequence of evolutionary events that lead to this diversity.

Here we investigate the role of the BMP pathway in DV patterning of the hemiptera *Rhodnius prolixus*, a vector of Chagas disease. *Rhodnius prolixus* has a sequenced genome [12] and well-established protocols for gene knockdown (KD) by RNAi [13]. Previously, we have shown that the Toll pathway is required for the expression of the ventral markers *twist* and *snail*, and development of the ventral mesoderm [14]. The current manuscript expands this analysis, by investigating the respective contribution of the Toll and BMP pathways for embryonic DV patterning. We show that *R. prolixus* relies mostly on BMPs for DV patterning, as shown for another hemiptera, *Oncopeltus fasciatus* [9]. This supports the hypothesis of a major role for BMPs in DV patterning at the basis of Hemiptera. Surprisingly, however, *R. prolixus* Sog and Twisted gastrulation (Tsg) extracellular modulators do not antagonize BMPs as established for general DV patterning in insect species, including *O. fasciatus* [9,15–17], but rather perform a positive role to establish graded BMP activity along the DV axis. Added to the lack of *sog* homologues in wasps [8] and crickets genomes [7], this diversity in the role of BMP modulators suggests that the mechanisms driving insect DV patterning are more complex than previously suspected.

## 2. Results

### 2.1. BMPs exert a central role to pattern the embryonic DV axis in *Rhodnius prolixus*

**Figure 1.**
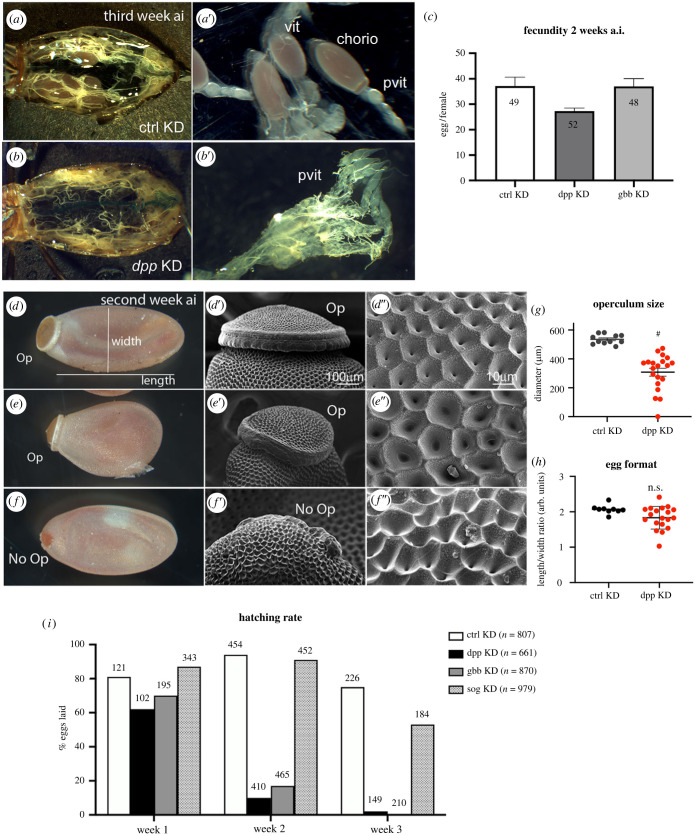
Maternal *dpp* controls oogenesis and eggshell morphogenesis in *Rhodnius prolixus*. (*a–c*) Oogenesis phenotypes resulting from parental knockdown (KD) with ds*Mal* (control; *a,a*′), and ds*dpp* (*b,b*′) (2 µg ul^−1^). Three weeks after blood ingestion (*a*.i), oocytes from *Rp-dpp* KD are not detected through the abdomen (*b*) as compared to controls (*a*). Ovaries dissected three weeks *a*.i. (*a*′,*b*′) show that ds*dpp* oocytes do not develop beyond vitellogenesis (*b*′). This is consistent with the tendency of a female fecundity drop initiated during the second week *a*.i. *n* refers to number of females analysed in two independent experiments (*c*). (*d–h*) Eggshell phenotypes resulting from KD with dsMal (d) or ds*dpp* (*e,f*) viewed with light microscopy (d–f) and scanning electron microscopy (*d*′–*f*′, *d*″–*f*″). *Rp*-*dpp* KD results in 36% of eggs with morphological alterations such as shortened eggs (e) and/or eggs displaying smaller operculi (*e,f*, *e*′*,f*′). In eggs lacking the operculum, alterations in eggshell ultrastructure are also observed (*f*″). (*g,h*) Quantification of eggshell phenotypes: (*g*) operculum size quantification; (*h*) Egg shape quantification defined by measuring the long (length) and short (width) axis. #*p* < 0.0001, Student *t*-test. (*l*) Embryonic viability with time after blood ingestion. Representative experiment with all conditions performed in parallel. Note the progressive loss of viability with the knockdown of BMP-encoding genes *Rp*-*dpp* and *Rp-gbb*. Even using a large amount of females, the number of *Rp-dpp* KD eggs for analysis is smallest due to the loss of fecundity shown in *b,c*. Numbers above bars correspond to the number of eggs laid at each time point. Pvit, pre-vitellogenic oocytes; vit, vitellogenic oocytes; chorio, choriogenic oocytes; Op, operculum.

To explore the roles for BMPs in *R. prolixus* embryonic patterning, we performed functional analysis for the two BMP homologues we formerly identified [12] by parental RNA interference (pRNAi) [13]. Knockdown (KD) for *decapentaplegic* (*dpp*) resulted in loss of fecundity, particularly after the third week after blood feeding, while KD for either *dpp* or *glass bottom boat* (*gbb*) loci resulted in loss of embryonic viability (figure 1). *Rp-dpp* KD, but not *Rp-gbb* KD, affects oogenesis, resulting in ovarioles that halt their maturation before vitellogenesis (figure 1*a,b*). Eggshell defects are also observed with the maternal injection of *Rp-*dpp dsRNA, where unhatched eggs often display small or absent operculi (figure 1*d–h*). A significant percentage of the eggs display eggshell defects (32%), with a variable loss of the operculum and collar, shortened AP axis and altered cuticular structure. Despite these defects, it is still possible to identify the dorsal and ventral sides of the eggs, indicating that other pathways are responsible for defining the oocyte DV axis. Expression during all stages of oogenesis [14,18] is consistent with the oogenesis and eggshell defects resulting from *Rp-dpp* knockdown.

**Figure 2.**
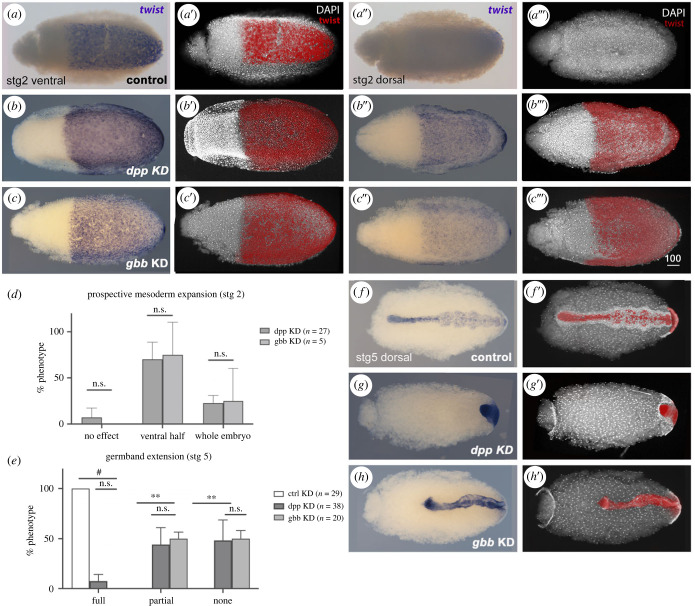
BMPs pattern the embryonic DV axis in *R. prolixus.* (*a-f*) *Rp-twi* expression in stage 2 (*a–c*) or stage 5 (*f–h*) embryos resulting from control (*a,f*), *Rp-dpp* (*b,g*) and *Rp*-*gbb* (*c,h*) parental KD, in ventral (*a,b,c*; *a*′,*b*′,*c*′) and dorsal (*a*′,*b*′,*c*′; *a*″,*b*″,*c*″; *f*,*g*,*h*; *f*′,*g*′,*h*′) views. (*d*) Quantification of stage 2 *Rp-dpp* and *Rp-gbb* KD shows that most embryos display an extended presumptive mesoderm marked by *Rp-twi*, either entirely along the DV axis (whole embryo), indicating complete ventralization, or extending only along the ventral half of the embryo (ventral half). (*e*) Quantification of stage 5 embryos with wild-type germband extension (no effect), partially extended germband (partially extended, *Rp-twi* domain as in (*h*) or halted germband extension (none, as in *g*) phenotypes, resulting from KD with ds*Mal* (*f*,*f*′ control), ds*dpp* (*g*,*g*′) and ds*gbb* (*h*,*h*′) as dorsal views of the egg. False colour of the *Rp-twi* expression domain in red is used to place the domain respective to the egg and to the germband, highlighted by DAPI nuclear stains (*a*′–*c*′, *a*″′–*c*″′, *f*′,*g*′). Graphs show mean and SEM from two independent experiments. 1-way ANOVA (*d*) and 1-way ANOVA with Tukey test (*e*). #*p* < 0.0001, ** *p* < 0.005, n.s. = not significant.

In addition to oogenesis, *Rp-dpp* and *Rp-gbb* are expressed during embryogenesis [14,19], with a diffuse expression pattern in blastoderm embryos (stage 2, electronic supplementary material, figure S1). In terms of embryo morphogenesis, both *Rp-dpp* and *Rp-gbb* parental KD lead to loss of viability (figure 1*i*) and embryos with halted germ band extension. To verify whether this halted development results from incorrect DV patterning, we performed *in situ* hybridization for genes differentially expressed along the dorsal–ventral axis (DV markers; figure 2 and electronic supplementary material, figure S2). Knockdown for either BMP-encoding loci resulted in dorsal expansion of ventral *Rp-twi* expression (figure 2*a–c*). In *Rp-dpp* KD, *Rp-twi* expression either expanded partially to fill the ventral half of the embryo (57%, *n* = 21) or expanded along the entire DV axis (29%), suggesting that this difference stems from variable effectiveness of the knockdown (figure 2*d*). In *Rp-gbb* KD, a similar pattern was observed. After germband extension stages (stage 5), a significant fraction of *Rp-dpp* and *Rp-gbb* KD embryos displayed a mass of posterior tissue that did not undergo ingression and expressed *Rp-twi* (figure 2*e–h*). Among the few embryos that did gastrulate, extension of the germband was partial. In these embryos, the *Rp-twi* expression domain was expanded in relation to the control (figure 2*h*). Stage 5 *Rp-soxNeuro* (*soxN*) expression, that marks a ventral row of neuroblasts on each side of the germband midline in control embryos, is irregularly expanded in *Rp-dpp* KD and *Rp-gbb* KD embryos (electronic supplementary material, figure S2). The results above indicate that loss of *R. prolixus* BMP function results in loss of dorsal territories and expansion of ventral and/or lateral gene expression in the early blastoderm, which impairs correct DV patterning and the resulting gastrulation process.

**Figure 3.**
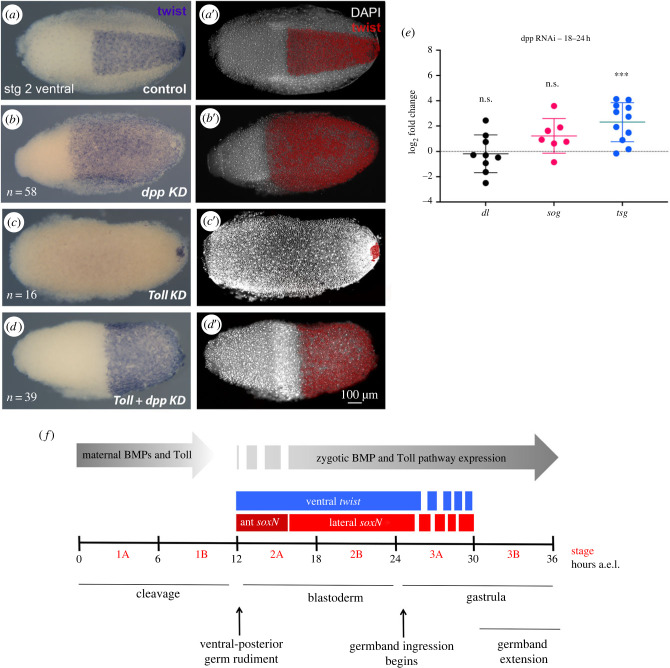
*Rp-dpp* KD reverts *Rp-Toll* KD loss of ventral gene expression. (*a–d*) *Rp*-*twi* expression in stage 2 embryos resulting from control (*a*-*a*′), Rp-*dpp* (1 µg, *b*.,*b*′), Rp-*Toll* (2 µg, *c*,*c*′) and Rp-*dpp* *+* *Rp-Toll* (1 µg + 2 µg, *d*,*d*′) parental KD, in light microscopy (*a–d*) or false colour (red) over nuclear DAPI stain (*a*′–*d*′) ventral views. (*e*) Quantitative Rt-PCR showing that knockdown for *Rp-dpp* significantly increases *Rp-tsg* expression and shows a tendency to increase *Rp-sog,* but not *Rp-dl*. Two-tailed *t* and Wilcoxon test, ***p < 0.005. (*f*) Temporal progression of morphogenesis and gene expression during early *R. prolixus* embryogenesis. The DV markers Rp-*twi* and Rp-*soxN* initiate zygotic expression during establishment of the embryonic blastoderm, when nuclei have already reached the periphery. The anterior *soxN* domain (ant *soxN*) is observed slightly before the lateral stripe, and at the same time as ventral *twi*. Ventral and lateral expression of these genes in the presumptive mesoderm and neuroectodermal territories is progressively altered as germband ingression proceeds. The effects of maternal Toll elements and BMPs are observed during cleavage stages, indicating the transfer of protein to the oocyte and future embryo. The expression levels of Toll pathway elements increase at stage 2A, while *Rp-dpp* expression levels increase after stage 2B, indication zygotic expression.

In addition to BMPs, Toll signals contribute to insect DV patterning, with the relative contribution of each pathway varying among different insect orders [20]. To investigate the relative contribution of the BMP and Toll pathways for *R. prolixus* embryonic DV patterning, we performed double knockdowns for *Rp-Tl* and *Rp-dpp* (figure 3). *Rp-dpp* KD and *Rp-Tl* KD result in opposite effects on DV patterning: in *Rp-dpp* KD, *Rp-twi* expression is expanded dorsally (figure 3*a,b*); in *Rp-Tl* KD the ventral *Rp-twi* domain is lost (figure 3*c*), consistent with the loss of mesodermal tissue and decrease in *Rp-twi* and *Rp-sna* expression previously reported for *dorsal* (*dl*) KD [14]. Double *Rp-Tl* *+* *Rp-dpp* KD resulted in expanded *Rp-twi* expression, similar to the pattern displayed by *Rp-dpp* KD alone (figure 3*d*). The DV phenotypes described above for *R. prolixus* are equivalent to those displayed by another hemiptera, *Oncopeltus fasciatus* [9]: *Of*-*twi* expression expands along the DV axis as a result of *Of-dpp* single and *Of-Tl* + *Of-dpp* double KDs, while *Of-twi* expression is lost in the *Of-Toll* parental KD. This reinforces the idea that Hemiptera rely to a greater extent on BMP signals for DV patterning instead of a major role for Toll as shown for Diptera. Other parts of the gene regulatory network (GRN) involving the BMP and Toll pathways in *O. fasciatus* and *R. prolixus* are also quite similar. In *O. fasciatus*, *dpp* inhibits *sog,* since *Of-dpp* KD results in dorsal expansion of *sog* expression [9]. In *R. prolixus*, *Rp*-*dpp* KD leads to an increase in the levels of *Rp*-*tsg* and a tendency to increase *Rp*-*sog*, which encode-conserved BMP modulators (figure 3*e*). Considering *Rp*-*twi* expansion in double *Toll* and *dpp* KDs for both species, it appears that neither species requires *Toll* for *twi* expression. Additionally, both *Of-dl* and *Rp-dl* favour *sog* expression [9,14] suggesting that *R. prolixus* Toll signals may act as suggested for *O. fasciatus Toll* to polarize *sog*. Further parts of the GRN, particularly involving the relationship between *Rp*-*sog* and *Rp*-*dpp*, in these two species warrants further investigation, as shown below.

In addition to DV patterning, we have previously shown that *Rp-Tl* also controls AP patterning of the *R. prolixus* embryo [14]. Interestingly, the anterior placement of the germband observed in *Rp-Tl* KD post-gastrula embryos was entirely reverted by *Rp-dpp* KD (electronic supplementary material, figure S3), suggesting that BMPs also control AP patterning. Since anterior placement of the germband was seen in blastoderm stage (stage 2) *Rp-Toll* KD embryos [14], errors in germband placement may be at least partially due to defective early AP patterning events. However, it is unlikely that Rp-Tl acts by inhibiting *Rp*-*dpp* expression in this context, since *Rp-dorsal* KD does not alter *Rp-dpp* mRNA levels [14]. A role for Rp-Tl in positioning of the germband along the AP axis is also reinforced by the anterior expression of *Rp-cactus (Rp-cact)* during early blastoderm stages (electronic supplementary material, figure S4). In *Drosophila*, *cactus* restricts the action of Dorsal to ventral regions of the embryo, enabling *zerknult* expression and induction of the dorsal amnioserosa [21]. In short-gem insects such as the beetle T. *castaneum* and the milkweed bug *O. fasciatus* [22,23], no role for *cactus* in defining the extraembryonic serosa has been reported [9,11]. However, in these insects the cells that will give rise to extraembryonic tissue are easily identified by the greater spacing between nuclei in the anterior region that gives rise to the serosa when compared with the nuclear spacing in the posterior ventral region that gives rise to the germband. In *R. prolixus* (electronic supplementary material, figure S5) this pattern is also observed, suggesting that the anteriorly localized nuclei give rise to the extraembryonic serosal tissue. Thus, it is conceivable that anterior *Rp-cact* expression restricts Rp-Toll signals posteriorly to allow anterior placement of the serosa. Unfortunately, this hypothesis cannot be tested directly at this time, since *Rp-cact* dsRNA injections in the female abdomen result in halted oogenesis [14].

### 2.2. Global effects of maternal *Rp-dpp* on early embryonic gene expression

To better understand the respective contributions of *Rp-dpp* and *Rp-Toll* to early embryonic pre-patterns, we decided to investigate whether the effects resulting from *Rp-dpp* KD were due to altered maternal or zygotic *Rp-dpp*. *Rp-dpp* is expressed at high levels during oogenesis, from pre-vitellogenic to chorionic stages [14,18,24]. During embryogenesis, early cleavage stages are practically devoid of *Rp-dpp* expression and high levels of *Rp-dpp* mRNA are only detected by RT-qPCR after stage 2B (18–24 h a.e.l., late blastoderm), with highest levels observed during stage 3A (gastrula [14]). Therefore, most zygotic *Rp-dpp* is expressed after the first effects of parental *Rp-dpp* KD on *Rp-twi* expression are observed (12–18 h a.e.l., stage 2A or early blastula, figure 2). This indicates that the earliest altered gene expression patterns are a result of maternal Dpp signals. These could result from an indirect effect of *Rp-dpp* expression during oogenesis or from maternal Rp-Dpp protein deposited in embryos that directly affects early embryogenesis. It is important to recall that the parental *Rp-dpp* KD leads to loss of *Rp-dpp* (electronic supplementary material, figure S1f), an increase in *Rp*-*tsg* and a tendency to increase *Rp*-*sog* expression during stage 2 (figure 3*e*). These effects could result either from direct interference on zygotic *Rp-dpp* messages and/or from impairing a positive feedback from maternal to zygotic *Rp-dpp*.

To test whether maternal *Rp-dpp* controls gene expression during the earliest stages of embryogenesis, we performed parental RNAi for *Rp-dpp* or an unrelated dsRNA as a control (dsMAL) and collected the resulting embryos for transcriptome RNA sequencing. Stage 1A (cleavage stage) embryos were used (0–6 h embryos) to avoid interference from zygotic *Rp-dpp* expression. Comparison of ds*MAL* and ds*dpp* KD resulting transcripts showed that 645 genes were downregulated (log fold change < −1.5) upon Rp-*dpp* KD and 28 were upregulated (log fold change greater than 1.5) (electronic supplementary material, figure S6*a–b*), showing that the maternal Dpp pathway acts mainly as an activator in this context. Although *Rp-dpp* (RPRC000401) showed very low expression levels in 0–6 h embryos, confirming previous report [14], we were still able to verify downregulated *Rp-dpp* transcripts after *Rp-dpp* KD (log fold change = −8,2; electronic supplementary material, figure S6*c* and table S3). Interestingly, several genes reported as important for stem cell maintenance, such as Lin-28 [25], were downregulated as a result of *Rp-dpp* RNAi (electronic supplementary material, table S3 and figure S6*d*). Noteworthy, genes used as markers for DV territories (such as *twi* and *soxN*) are not present in this dataset, since these genes are only expressed during blastoderm stages (> stage 2, or greater than 12 h embryogenesis). The large number of genes downregulated upon *Rp-dpp* KD when compared with the control suggests that maternal BMP signals are indeed capable of regulating early embryonic gene expression. Importantly, *Rp-gbb, Rp-dorsal* and *Rp-Toll* mRNA levels did not change upon *Rp-dpp* KD during the 0–6 h cleavage stage, suggesting that the Toll and BMP pathways function in parallel to establish the initial patterning events in the embryo.

### 2.3. Extracellular BMP modulators Sog and Tsg pattern the ventral half of the *Rhodnius prolixus* embryo

**Figure 4.**
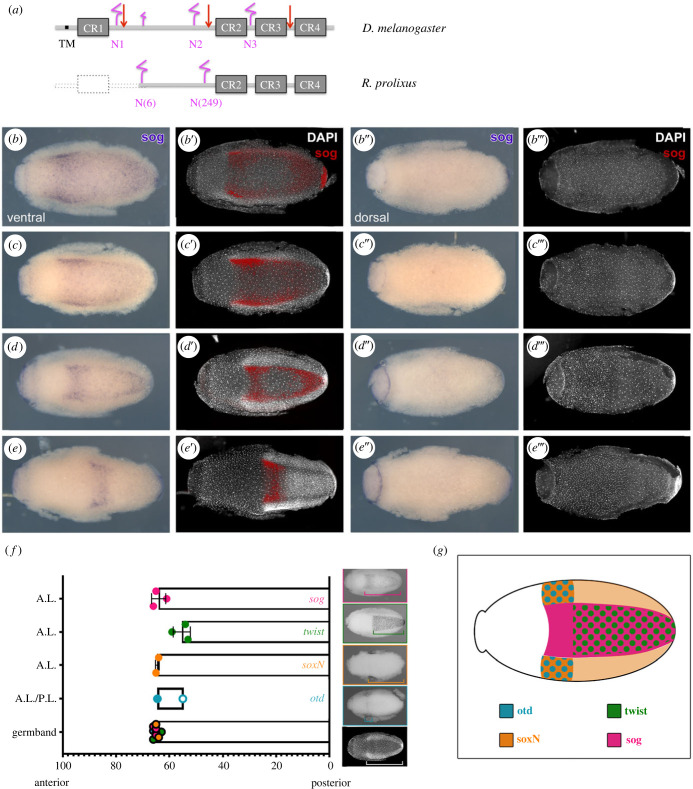
*short gastrulation* is expressed in ventral domains of the early *R. prolixus* embryo. (*a*) Partial Rp-Sog, compared to Dm-Sog, showing the four conserved cystein-rich (CR) and stem regions, as well as putative glycosylation sites (pink). Protein domains and placement of glycosylation sites were predicted based on sequence alignment with several insect Sog proteins (electronic supplementary material, figure S7 and table S4). *Rp-sog* sequence suggests missing 5′-region in the published genome. (*b–e*) *Rp-sog* expression pattern in the wild type, from the early blastoderm (stage 2A) to germband formation (stage 3), in ventral views alone (*b–e*) and in false colour (red) relative to nuclear staining (*b*′–*e*′) or dorsal views (*b*″–*e*″ and *b*″′–*e*″′). (*f*) Placement of the anterior limit of Rp-*sog* expression relative to the anterior limits of *Rp-otd, Rp-soxN* and *Rp-twi*. The anterior limit of the germband was measured from DAPI stainings, in order to ensure perfect equivalence of developmental stage between the different embryos. Colours in circles denote embryos where *in situ* hybridization was performed for *Rp-sog* (pink), *Rp-otd* (blue), *Rp-soxN* (orange) and *Rp-twi* (green). A.L. = anterior limit of expression. P.L. = posterior limit of expression respective to the posterior of the egg, defined as 0% egg length. Brackets on side figures illustrate the size of the domains measured (brackets). (*g*) Relative domains of gene expression for *Rp-sog, Rp-otd, Rp-soxN* and *Rp-twi,* based on measurements in F.

The establishment of graded BMP signals depends on the activity of extracellular modulators such as Sog, Crossveinless/Tsg (Tsg) and Crossveinless2 (Cv2) proteins, that bind to and inhibit BMPs but are also able to deliver BMP proteins at a distance from the production source [1]. *Rhodnius prolixus* displays one *sog*, one *cv*/*tsg* and one *cv2* homologue [12]. Three cystein-rich domains (CRs) were identified in Rp-Sog, as well as a stem region that harbours conserved putative glycosylation sites (figure 4*a*; electronic supplementary material, figure S7 and table S4). In the light of the high conservation of Sog structure among insects, including Hemiptera, it is possible that the *Rp-sog* sequence is incomplete and presents a first CR domain that is yet to be identified. The zygotic pattern of *Rp-sog* expression is highly dynamic (figure 4*b–e*), starting with ventral expression that becomes progressively strongest at the border of the *twi*-expressing and *soxN*-expressing cells, probably coincident with *Rp-single minded* (*Rp-sim*) expression in the mesectoderm (figure 4*d*, cf. 5*f*). No overlap with the *soxN*-positive lateral neuroectodermal cells is apparent (figure 4*g*). Later, expression resolves to a strong anterior band (figure 4*e*). This band is in great part anterior to the anterior part of the *twist* domain and extends as much as the anterior *orthodenticle* (*otd*) domain (figure 4*f,g*), suggesting that *Rp-sog* may also be involved in the development of head structures. Contrary to the *Rp-sog* expression pattern, we were unable to identify localized expression for *Rp-cv/tsg*. *in situ* hybridization for *Rp-cv2* was unsuccessful, despite all our efforts.

**Figure 5.**
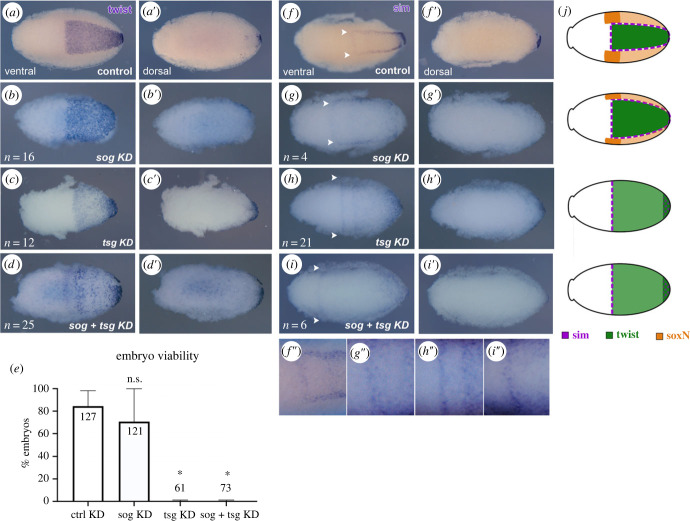
BMP antagonists pattern the ventral part of embryonic DV axis. *Rp-twi* expression in stage 2 embryos with knockdown for control (*a*), *Rp-sog* (*b*), *Rp-tsg* (*c*) and *Rp-sog* *+* *Rp-tsg* (*d*) in ventral (*a–d*) and dorsal (*a*′–*d*′) views. (*e*) Embryonic viability resulting from knockdown for control, *Rp-sog, Rp-tsg* and *Rp-sog* *+* *Rp-tsg.* Graph show mean and SEM. One way ANOVA with Tukey test, *p < 0.01, ns = not significant. (*f–i*) *Rp-sim* expression in stage 2 embryos with knockdown for control (*f*), *Rp-sog* (*g*), *Rp-tsg* (*h*) and *Rp-sog* *+* *Rp-tsg* (*i*) in ventral (*f–i*) and dorsal (*f*′–*i*′) views*. f*″–*i*″) Detail of anterior *Rp-sim* expression, showing that it expands laterally in the KDs. Arrows in *f–i* indicate the lateral and anterior limits of the *Rp-sim* domain. The increased distance between lateral *Rp-sim* expression domains follows the expansion of the *Rp-twi* domain in KD for *Rp-sog* and *Rp-tsg.* (*j*) Scheme depicting expression patterns shown in *a–d* and (*f–i*).

To investigate the role of extracellular BMP modulators on *R. pro*lixus DV patterning, we performed single and double knockdowns for their encoding loci. Maternal dsRNA injections for *Rp-sog* resulted in a clear expansion of the ventral *Rp-twi* domain, as observed for BMP knockdowns (figure 5*a,b*). Unlike the expansion observed for *Rp-dpp* and *Rp-gbb* KDs, however, in *Rp-sog* KD expansion of the *Rp-twi* domain was restricted to the ventral half of the embryo. *Rp-tsg* KD (figure 5*c*) and *Rp-sog* + *Rp-tsg* double KD (2 µg + 2 µg, figure 5*d*) resulted in a similar expansion of the *Rp*-*twi* domain along the ventral half of the embryo. This indicates that *Rp-sog* and *Rp-tsg* act together to pattern ventral domains of the embryo. Accordingly, parental RNA interference for *Rp-sog* *+* *Rp-tsg* double KD led to a significant drop in embryonic viability (figure 5*e*).

**Figure 6.**
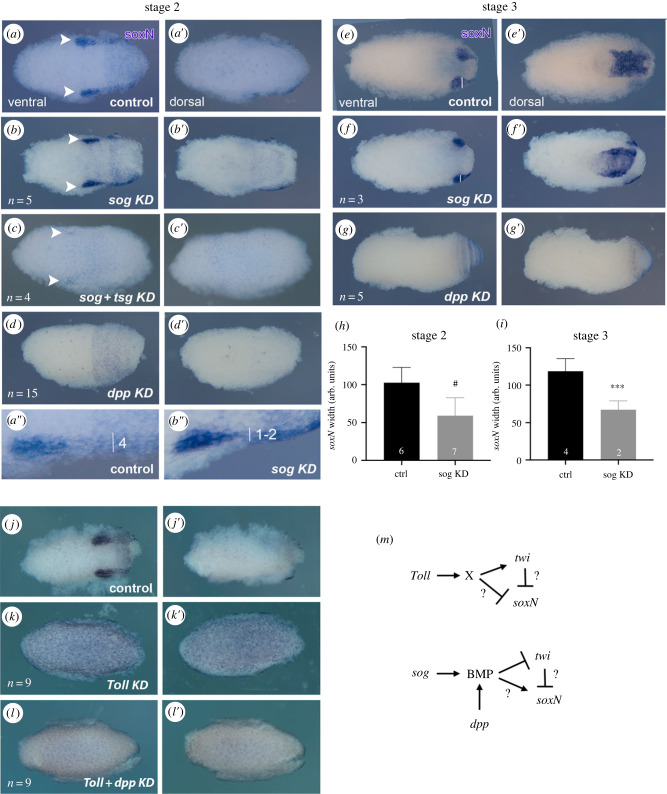
Knockdown for *R. prolixus* BMP and BMP modulators exhibit similar effects on the neuroectodermal domain. (*a–d*) *Rp-soxN* expression in stage 2 embryos with knockdown for control (*a*), *Rp-sog* (*b*), *Rp-sog* *+* *Rp-tsg* (*c*) and *Rp-dpp* (*d*) in ventral (*a–d*) and dorsal (*a*′–*d*′) views. The embryo in *c* is slightly younger than *a,b*, defined by the anterior limit of the germband (arrowheads in *a,b,c*). *a*″,*b*″) Detail of the anterior part of the lateral *Rp-soxN* domain, showing that it is around four cells wide in control (*a*″) and one-two cells wide in *Rp-sog* KD (*b″*). (*e–g*) *Rp-soxN* expression in stage 3B embryos with knockdown for control (*e*), *Rp-sog* (*f*), and *Rp-dpp* (*g*) in ventral (*e–g*) and dorsal (*e*′–*g*′) views. (*h,i*) Using embryos of the exact same stage of development, the lateral Rp-*soxN* was quantified in stage 2 (*h*) and stage 3 (*i*) control and *Rp-sog* KD embryos. Student *t*-test, *** *p* < 0.005, # *p* < 0.0001. (*j–l*) The antagonic effects of *Rp-dpp* and *Rp-Toll* on *Rp-soxN* expression are shown in control (*j*), *Rp-Toll* (*k*) and *Rp-Toll* *+* *Rp-dpp* (*l*) KDs, in ventral (*j–l*) and dorsal (*j′*–*l′*) views. (*m*) Proposed effect for Toll and BMP pathways on mesodermal *Rp-twi* and neuroectodermal *Rp-soxN* expression.

Since *Rp-sim* and *Rp-soxN* delineate the *Rp-twi* domain in wild-type embryos, we investigated whether their expression domains were modified in *Rp-sog* KD. In fact, the *Rp-sim* domain is displaced dorsally (figure 5*f,g*), suggesting that the mesectoderm is formed in these embryos, but that it is dorsally displaced to localize at the new border of the expanded *Rp-twi* domain. In *Rp-tsg* KD and *Rp-sog* + *Rp-tsg* KD the anterior border of *Rp-sim* is expanded entirely at the ventral half of the embryo, making it difficult to define whether the *Rp-sim* at the *Rp-twi* border is displaced or simply abrogated (figure 5*h,i*). *Rp-sog* KD embryos also showed altered neuroectodermal gene expression. *Rp-soxN* is expressed as a strong lateral band in the prospective neuroectoderm and weakly as a broad ventral territory (figure 6*a*). In *Rp-sog* KD, the lateral band that marks the neuroectoderm adjacent to each side of the *Rp-twi* domain is displaced dorsally (figure 6*b*). The lateral *Rp-soxN* domain is also thinner in *Rp-sog* KD than in control embryos, indicative of loss of ventral-lateral regions of the neuroectoderm (figure 6*h,i*). On the other hand, *Rp-sog* + *Rp-tsg* KD leads to loss of strong *Rp-soxN* expression in the lateral domains adjacent to *Rp-twi*, with weak expression remaining along the ventral germband (figure 6*c*). Expansion of weak *Rp-soxN* is seen only in the ventral half of these embryos. Based on the short distance between nuclei (electronic supplementary material, figure S5), strong lateral *Rp-soxN* expression likely marks the lateral neuroectoderm, while ventral *Rp-soxN* coincides with *Rp-twi* in the mesodermal anlage. Note that the strong lateral *Rp-soxN* domain is absent in *Rp-sog* *+* *Rp-tsg* KD. Surprisingly, this is very similar to the pattern of *Rp-dpp* KD (figure 6*d*). In embryos that reach the germband extension stages, a greater distance between the two lateral *Rp-soxN* domains is seen in *Rp-sog* KD compared to control embryos (figure 6*e,f*). In *Rp-dpp* KD, the germband does not extend and *Rp-soxN* appears along a ring at the posterior end of the embryo (figure 6*g*). The loss of lateral *Rp-soxN* expression agrees with the expanded *Rp-twi* domain in *Rp-sog* *+* *Rp-tsg* KD and suggests that *sog* and *tsg* are required to subdivide the ventral half of the embryo in presumptive ventral mesoderm (*Rp-twi*) and lateral neuroectoderm (*Rp-soxN*) (figures 5*d* and 6*c*). Unfortunately, we were unable to investigate the effects of *Rp-sog* and *Rp-tsg* KDs on dorsal domains of the embryo due to the lack of suitable dorsal–lateral or dorsal markers.

Based on the results above, positioning of the lateral neuroectodermal domain and the extent of the ventral mesodermal territory seem to rely greatly on BMP signals rather than on the Toll pathway. This is consistent with the pattern of *Rp-soxN* expression in *Rp-Toll* KD, where *Rp-soxN* expression expands along the entire DV axis (figure 6*k*). *Rp-soxN* expansion is reverted by the concomitant loss of BMP signals (*Rp-Toll* *+* *Rp-dpp* KD, figure 6*l*), in agreement with the loss of *Rp-twi* expression and *Rp-twi* expansion in *Rp-Toll* and *Rp-dpp* KDs, respectively (figure 3).

It is important to point out that Sog/Chordin and Tsg homologues bind BMPs to either inhibit BMPs from receptor binding or to control BMP spatial distribution away or against a BMP source [20,26]. Taking into account that *Rp-sog, Rp*-*tsg* and *Rp*-*dpp* KD all result in the expansion of the ventral marker *Rp*-*twi* and loss of the neuroectodermal marker *Rp*-*soxN*, we suggest that Rp-Sog and Rp-Tsg proteins perform mainly a positive role to modify BMP distribution along the *R. prolixus* DV axis, away from the source of ventral Rp-Sog (figure 6*m*). Graded BMP signals set up by dorsal shuttling of BMPs would then subdivide the ventral half of the embryo in a *Rp-twi* expressing mesodermal domain (low BMP) and lateral *Rp-soxN* neuroectoderm territory (intermediate BMP).

## 3. Discussion

### 3.1. Maternal BMP signals control oogenesis and embryonic patterning

BMPs perform several roles during insect oogenesis and embryogenesis. The oogenesis defects for *Rp*-*dpp* KD here described are reminiscent of defects reported for *Drosophila*. *Drosophila dpp* is expressed in the germarium to control germline stem cell differentiation [27] and during mid-oogenesis [28]. At this later stage, the expression in dorsal–anterior follicle cells that cover the oocyte is fundamental to pattern the future chorion that will cover the egg in drosophilids [29]. Loss of function of *D. melanogaster dpp* or of the BMP receptor *tkv* results in a decrease in operculum size at the dorsal–anterior region of the eggshell [28,30], while *dpp* overexpression results in loss of appendages and increase in the operculum [28,31]. The eggshell cap and collar perform a function that is equivalent to the operculum in *Drosophila*, that is, both define the region from where the animal hatches at the larval (*Drosophila*) and first instar (*Rhodnius*) stage. This may suggest a conserved function for BMPs in insect eggshell morphogenesis [32]. As here suggested for *R. prolixus*, loss of *D. melanogaster* BMP function during mid-oogenesis does not affect the egg DV axis, since the egg DV pattern is maintained and the expression of *pipe* and *gurken*, upstream elements of the Toll and EGFR pathways that define the follicle DV axis, are unaltered [33]. It is yet to be defined whether EGF signaling provides the symmetry-breaking event in the *R. prolixus* egg as it does in *D. melanogaster* and in other species [7,34].

In addition to the effects of BMPs on oogenesis and eggshell morphogenesis, our data suggests that BMPs pattern the embryo along the AP and DV axis. We are tempted to suggest that an early effect of maternal Dpp and Toll sets an anterior to posterior gradient of gene expression required to set the placement of the dorsal–anterior extraembryonic serosa versus ventral–posterior germband, consistent with the anterior placement of the germband in *Rp-Toll* KD and recovery of posterior germband placement in the *Rp-Toll* *+* *Rp-dpp* double KD. A role for Toll in AP patterning has been proposed in *R. prolixus* [14], *O. fasciatus* [9] and *Apis mellifera* [35]. In the bee, *Am*-*cact* is enriched to the anterior pole of the oocyte and cellularized embryo, as herein shown for *Rp-cact*. Additionally, *Am*-*dpp* expression is required for anterior-dorsal *Am*-*zen* expression and serosal patterning, Early *Rp*-*cact* expression in the anterior blastoderm may help to inhibit anterior Rp-Dl function, restricting Toll signals to posterior blastoderm nuclei. However, from our RNAseq analysis (0–6 h) we can infer that maternally provided *Rp-cact* is not transcriptionally regulated by *Rp*-*dpp*, since no variation in expression between control and *Rp-dpp* KD embryos was observed. Therefore, it is unclear how *Rp-dpp* KD is able to revert the anterior placement of the germband generated by *Rp-Toll* KD. An extended analysis of *Rp-dpp* targets in early embryonic stages will be required to understand these effects.

Importantly, the anteriorly localized *Rp*-*cact* expression shows no DV axis asymmetry during early embryogenesis, suggesting that an AP factor regulates its expression. While this expression pattern is suggestive of a role in the establishment of extraembryonic tissue, whether *Rp-cact* actually performs a role to define these tissues is unclear. In *Drosophila*, maternal *cactus* enables *zen* expression in the amnioserosa by restricting the repressive action of Dorsal to ventral regions of the embryo l [36]. Drosophila *cactus* is also a zygotic target of Dorsal in the ventral mesoderm [37]. In the beetle *Tribolium castaneum*, *Tc*-*cact* is only zygotically transcribed and essential for the establishment of a feedback circuit, which terminates the nuclear Dorsal gradient during late blastoderm stages [11]. In another holometabolous insect species with long germ development, the wasp *Nasonia vitripennis, Nv*-*cact* expression is zygotic and restricted to a narrow stripe straddling the ventral midline [38,39]. In *O. fasciatus* (hemiptera) six *cact* paralogs exist, four of which are expressed during blastoderm stages [9]. Apparently, the most important orthologue for early embryonic development *Of-cact3* is expressed in a broad ventral domain encompassing 60–80% of the embryonic circumference with graded borders toward the dorsal side and its expression does not refine into a narrower domain as observed in *Tribolium*. Sachs *et al.* [9] assumed that the broad, weakly graded expression of *Of-cact3* in *O. fasciatus* reflects a flat Toll signalling gradient which extends from the ventral to the dorsal half of the embryo. Recent analysis of immune activation during embryogenesis in this hemiptera showed an upregulation of expression of another *cact* orthologue (*Of-cact1*) upon septic injury, probably in the serosa. This analysis demonstrates a sub-functionalization of at least two *cactus* paralogues, Of-*cact1* and Of-*cact3*, with one paralogue involved in DV axis formation and the other with an immune function in the serosa [40]. Lastly, in the orthoptera *Gryllus bimaculatus* [7] *cactus* is not expressed in the embryonic ventral side neither in the serosa and there is no evidence of zygotic feedback mechanisms modulating the Dorsal gradient. Thus, our data provides a new important phylogenetic reference species for the evolutionary analysis of *cactus* expression and function in insects. In contrast to *O. fasciatus*, [9], only one *cactus* orthologue has been identified in the *R. prolixus* genome. Since this single orthologue shows no DV asymmetry in expression and is only expressed in the prospective serosa during early developmental stages, our data favour a model where self-regulatory circuits modulated by *cactus* as observed in *Tribolium* DV axis are not present in *Rhodnius*. Thus, at least two hypotheses are plausible for *cactus* evolution in hemiptera. In the first hypothesis, the DV function of *cactus* observed in *Oncopeltus* was lost in the lineage that gave rise to *Rhodnius,* while the extraembryonic role was maintained. In the second hypothetical scenario, the ancestral role of *cactus* in hemiptera would be extraembryonic specification and the DV role of *cactus* was independently acquired in *Oncopeltus*. Further functional studies are required to reconcile these possibilities.

**Figure 7.**
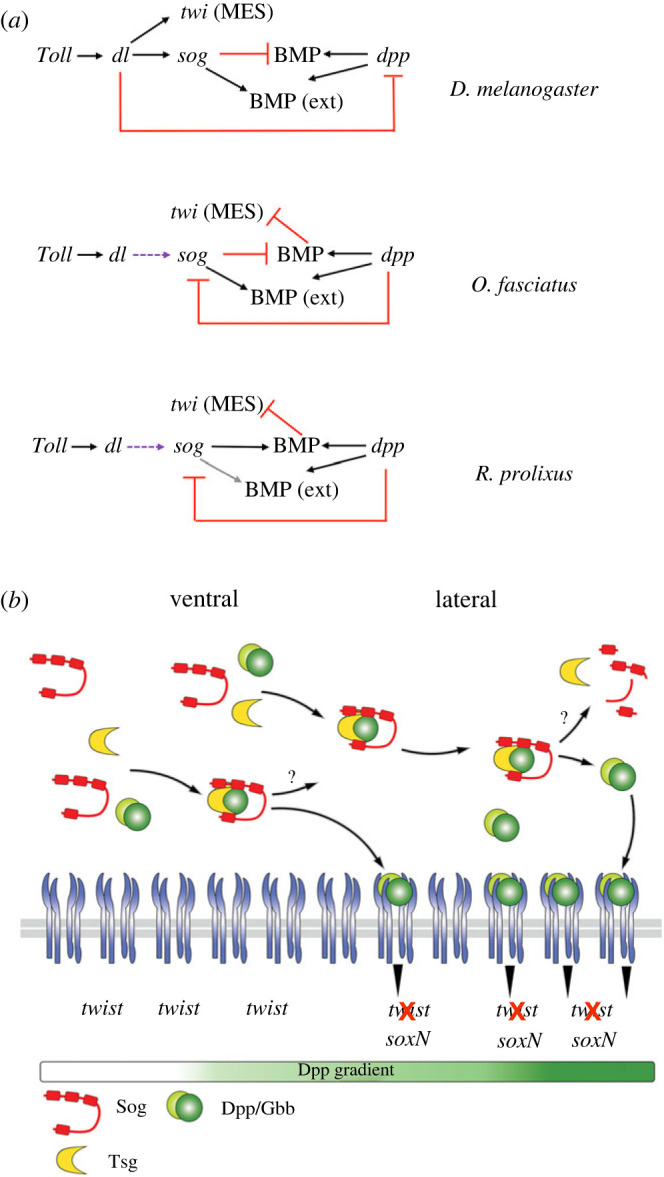
Model for the action of the Toll and BMP pathways in *R. prolixus* DV patterning. (*a*) The gene regulatory network (GRN) for the Toll and BMP pathways changes throughout insect evolution. In the first described GRN, *D. melanogaster* Toll induces a Dorsal nuclear gradient that activates *twi* expression in the mesoderm and *sog* expression in the neuroectoderm and inhibits *dpp* expression in these regions. BMPs (*dpp*) have a role restricted to defining dorsal domains of the embryo as the extraembryonic amnioserosa (Ext). Sog (and Tsg) shuttle BMPs dorsally for amnioserosa formation (arrow). In *O. fasciatius*, Toll only polarizes *sog* (purple arrow), while BMPs (*dpp*) are required to restrict *twi* expression to ventral regions. Loss of *twi* expression in *sog* KD indicates that Sog inhibits BMPs in the ventral domain, maintaining *twi* expression. Sog (and Tsg, not depicted) may also shuttle BMPs for induction of the serosa (Ext). In *R. prolixus*, Toll has a restricted role and BMPs perform a central role to define the DV axis as in *O. fasciatus*. However, differently from *O. fasciatus*, expansion of *twi* expression in *sog*, *tsg* and *dpp* KDs indicates that Sog (and Tsg) favour BMPs to restrict *twi* expression to the ventral prospective mesoderm. A role for Sog/Tsg to shuttle BMPs for serosa formation awaits the identification of dorsal markers (grey arrow). (*b*) Based on the formation of Sog/Tsg/BMP complexes shown for vertebrates and invertebrates, and a ventral source of Sog protein and on the KD phenotypes herein described, we propose that in *R. prolixus* a Sog + Tsg complex is generated ventrally and shuttles Dpp/Gbb heterodimers dorsally, allowing to attain appropriate levels of BMPs to inhibit *Rp-twi* and to favour neuroectodermal gene expression in the lateral region of the embryo. Mes, mesoderm; DE, dorsal ectoderm; Ser, Serosa.

Altogether, the restricted role of a Toll pathway element (*Rp-cact*), the complete reversion of Toll pathway KD DV phenotypes by *dpp* KD, and the strong effects of BMP pathway elements (*sog, tsg, dpp* and *gbb* KDs) on ventral and lateral gene expression (*twi*, *sim* and *soxN*) are in agreement with a minor role of Toll signals in *R. prolixus* DV patterning, as compared to BMPs. Despite the changing GRNs involving Toll and BMP pathway elements in DV patterning (figure 7*a*), it is most likely that the network itself was set early in insect evolution, before the establishment on the Hemiptera order.

### 3.2. A broad effect of BMPs on *Rhodnius prolixus* embryonic DV patterning

The observation that *Rp-dpp* KD alters gene expression in cleavage stage embryos (Stage 1, 0–6 h development) indicates that at least some of the effects observed on early patterning are due to maternally expressed *Rp-dpp.* Maternal effects could be direct or indirect, with maternal Dpp protein transferred from oocytes acting directly to regulate embryonic events or indirectly, by regulating gene expression during oogenesis that would secondarily alter zygotic transcription.

With respect to early DV patterning, we propose that Gbb/Dpp heterodimers maintain mesodermal gene expression restricted to ventral territories, since with either *Rp-dpp* or *Rp-gbb* RNAi the *Rp-twi* expression domain is expanded along the entire DV axis. In addition, since *Rp*-*soxN* expression is lost in *Rp-dpp* or *Rp-gbb* RNAi, it is reasonable to suggest that intermediate levels of BMPs are required for Rp-*soxN* expression in the neuroectoderm, acting either directly or indirectly. Furthermore, the expansion of *Rp-twi* and loss of *Rp-soxN* expression following *Rp-dpp* KD is observed before significant levels of zygotic *Rp-dpp* mRNA are detected by RT-qPCR [14] (figure 3). Therefore it is most likely that a maternally provided Dpp/Gbb complex controls the initial *Rp-twi* and *Rp*-*soxN* DV pattern. Zygotic BMPs may either induce or reinforce DV patterning in the embryonic blastoderm, the latter characterizing a positive feedback loop between maternal and zygotic BMP provision. However, to clearly discriminate the respective roles of maternal versus zygotic *Rp-dpp* in DV patterning will require future experiments using embryonic instead of parental RNA interference.

Interestingly, previous analysis suggested that, in *Drosophila*, maternal Dpp protein is produced during oogenesis and delivered to the perivitelline space that covers the future embryo where it will impact ventral regions of the embryonic DV axis [30,33]. This ‘delayed induction’ of embryonic pattern by maternal proteins is well established for upstream elements of the Toll pathway in *Drosophila* [41]. A similar mechanism for maternal Dpp action could take place in *R. prolixus*. In several insects where BMPs are central players in defining the embryonic DV axis, it is not clear whether the parental RNAi used to uncover their role has affected maternal or zygotic components of BMP signalling. In *T. castaneum* embryonic injections of *Tc-dpp* or *Tc-Toll* RNAi leads to identical phenotypes to parental RNAi, showing that in this beetle zygotic patterning plays a major role for DV patterning [6,11]. However, maternal provision is the main source of BMPs that impacts embryonic DV patterning in *Nasonia vitripennis* and *Apis mellifera* [8,35]. In conclusion, it will be interesting to investigate whether maternal provision of *dpp* mRNA or protein is a conserved mechanism to pattern the insect embryonic DV axis.

### 3.3. The BMP modulators Sog and Tsg behave as agonists of BMP function in *Rhodnius prolixus* DV patterning

As morphogens, the range of BMPs' actions is dependent on their distribution and concentration along the tissue. Vertebrate Chordin, Twisted Gastrulation and Crossveinless 2, and insect Short gastrulation, Tsg and Cv2 homologues control BMP diffusion and consequent action range during vertebrate [42] and insect [21] DV patterning, mouse axial skeletal development [43,44] and *Drosophila* wing vein patterning [45–47]. Graded BMP activity has been detected along the DV axis of the *Xenopus* and *Drosophila* embryo, established by an inverse gradient of Chd and Sog protein, respectively [48]. Chd/Sog and Tsg proteins frequently act as a complex to inhibit the binding of BMPs to their receptors [49], and/or shuttle BMPs away from or against their source [49,50]. For instance, in the *Xenopus laevis* embryo Chd and Tsg interact as a complex to inhibit BMP signaling around the dorsally located Spemann organizer and establish a ventral-to-dorsal gradient of BMP activity [17,51,52]. A Chd-independent function for Tsg has also been reported [51]. In *Drosophila* DV patterning, Sog and Tsg inhibit Dpp autoactivation in the lateral neuroectoderm [53], and also shuttle Dpp against its source in the dorsal part of the embryo to concentrate a sharp field of Dpp activity required for amnioserosa formation [54]. A shuttling function for Sog has also been suggested in the induction of *Clogmia* and *Musca* dorsal extraembryonic tissue [5,55]. Likewise, *T. castaneum* Sog inhibits BMP function in the neuroectoderm and shuttles Dpp for serosa formation [6]. Interestingly, in this beetle, *tsg* also performs a *sog* independent pro-BMP function [15]. Therefore, a pro-BMP action of Sog and Tsg BMP modulators is regularly deployed to regulate the BMP action range and activity in insects.

While there are several examples of a pro-BMP activity of BMP modulators to induce the development of extraembryonic tissue, we have uncovered for the first time a pro-BMP function for Sog to pattern the ventral and lateral territories of the insect embryonic DV axis. Up to date, loss-of-function for insect *dpp* homologues lead to the expansion of lateral and/or ventral domains of gene expression that characterize the neuroectodermal and mesodermal territories. Contrarily, loss-of-function for *sog* homologues leads to loss of these same domains [6,9,56], suggesting that *sog* homologues antagonize BMPs in most insect DV patterning. Strikingly, loss-of-function for *R. prolixus sog* and *tsg* results in the expansion of ventral *Rp-twi*. This pro-BMP effect is limited to the ventral half of the embryo, since *Rp-sog* *+* *Rp-tsg* KD leads to expansion of *Rp-twi* and *Rp-sim* only in the ventral–lateral territories. *Rp-dpp*, on the other hand, is capable of inhibiting *Rp-twi* in dorsal and lateral regions of the DV axis, indicating that Sog and Tsg are not required for *Rp-twi* inhibition by *Rp-dpp* in the dorsal domain. Of note, we are yet unable to identify putative effects of Sog and Tsg on dorsal extraembryonic territories due to the lack of appropriate markers. Thus, we cannot ensure that *Rp-sog* and *Rp-tsg* do not have other effects to modulate BMP signals in the dorsal part of the embryo.

Based on *Rp-sog* expression in ventral cells of the germ band, that partially overlaps *Rp-twi* expression, we hypothesize that secreted Sog protein diffuses dorsally. As Sog protein forms complexes with Tsg and BMPs [20], Sog diffusion would allow shuttling of BMPs away from the ventral Sog source, allowing BMP levels to increase laterally. These intermediate BMP levels would then inhibit mesodermal genes (as *Rp*-*twi*) and allow neuroectodermal gene expression (as *Rp*-*soxN*), establishing the dorsal limit of the *Rp-twi* domain and defining the extent and location of the prospective lateral neuroectoderm (figure 7*b*). This hypothesis is in agreement with the dorsally displaced and thin band of *Rp-soxN* seen in *Rp-sog* KD, as compared to wild type.

In the *Drosophila* neuroectoderm and the chick neural tube, it has been shown that BMPs repress the expression of neural genes in a threshold-dependent manner [57], suggesting a conserved function for BMPs in patterning metazoan neural tissues. It will be interesting to investigate whether a graded effect for BMPs, by the action of Sog and Tsg, is required for subdivision of the kissing bug neuroectodermal domains once *in situ* hybridization for *vnd*, *ind*, *rho* and *msh* homologues are developed.

Considering a pro-BMP function for Sog and Tsg, we envision at least two mechanisms that could provide this effect: (i) Sog and Tsg shuttle BMPs dorsally, concentrating sufficient BMP protein in the lateral neuroectoderm to inhibit *Rp-twi* expression. With loss of Sog or Tsg, Dpp protein would dilute by ventral diffusion and would not achieve the threshold for *Rp-twi* inhibition in lateral and ventral domains; (ii) Tsg could facilitate binding of BMPs to their receptors to ensure *Rp-twi* inhibition in the lateral neuroectoderm, in parallel to dorsal BMP shuttling in a complex formed with Sog, as suggested for *T. castanuem* [15]. Based on qRT-PCR data, as BMP levels rise, they inhibit *Rp-tsg* and possibly *Rp-sog* expression, spatially limiting the action range of the BMP modulators. For each of the mechanisms suggested above, destruction of the Sog/Tsg/BMP complex for the delivery of BMP dimers to their receptors is necessary. This could be achieved by Tolloid (Tld) metalloproteases, which have been shown to cleave Sog/Chordin in deuterostomes and protostomes and deliver BMPs for receptor binding [references in 28]. We have previously identified *Rp*-*tld* [12] (RPRC002377). However, the functional significance of *Rp*-*tld* in *R. prolixus* embryonic patterning is yet to be established.

A series of extracellular elements modulate BMP function. In addition to the kernel extracellular modulators Sog/Tsg/Tld, components of the extracellular matrix such as collagen and proteoglycans control BMP diffusion throughout a tissue [58–60]. Consequently, assorted or graded BMP activity can be generated from uniform BMP expression. It is frequent to find contexts where Sog or Tsg are not deployed to generate non-uniform BMP activity. For instance, in the *Drosophila* wing imaginal disc, a gradient of Dpp protein is generated by the action of proteoglycans [61–63] and by interaction with BMP receptors [63,64]. This implies that the mechanisms used to generate graded BMP activity can be extremely diverse. Taking this diversity into account, the great disparity we observe between Sog and Tsg function in two hemiptera, *Rhodnius* and *Oncopeltus*, becomes more easily admissible. While both species seem to require Sog and Tsg to modulate Dpp function, *O. fasciatus* Sog/Tsg modulates BMP activity by antagonizing Dpp, while *R. prolixus* Sog/Tsg modulates BMPs by a pro-BMP effect. Furthermore, *Nasonia*, *Apis* and *Gryllus* do not require Sog for embryonic DV patterning [7,8,35], although the hymenoptera rely strongly on BMPs to subdivide the entire DV axis. Based on the data herein presented and on the inconsistent deployment of Sog and Tsg for insect embryonic DV patterning, it is tempting to suggest that the function of BMP modulators is less constrained than BMP function itself, allowing greater divergence of Sog function inside the insect clade.

## 4. Materials and methods

### 4.1. Insect rearing

*Rhodnius prolixus* rearing was performed at 28°C and 70–75% humidity, with animals fed on rabbit blood throughout all developmental stages. Technicians dedicated to the animal facility at the Institute of Medical Biochemistry (UFRJ) conducted all aspects related to rabbit husbandry under strict guidelines to ensure careful and consistent animal handling.

### 4.2. Functional analysis

RNA interference assays (RNAi) were used to study gene function. Double-stranded RNA was synthesized from DNA fragments generated by two rounds of PCR. For the first round, primers contained sequences to amplify specific products plus short (8 nucleotides) overhangs to the T7 Universal forward and reverse primers. For the second PCR, 2 µl of the first reaction were used as template for T7 universal forward and reverse primers. Primer pairs are listed in electronic supplementary material, table S1. For double-stranded RNA (dsRNA) synthesis, *in vitro* transcription followed, using the MEGAscript kit (Ambion), as per manufacturer instructions. For parental RNAi, 2 µl (unless stated otherwise), of each dsRNA (1 µg/μl) were injected in the abdomen of adult females, 3–5 days prior to blood feeding. Eggs were collected, counted, and the hatch rate defined after 20 days at 28°C, taking into account that wild-type embryogenesis lasts 14–15 days at this temperature. Since molting to second instars requires approximately 15 days following feeding, viability at this stage was defined 20 days after blood ingestion. Efficiency of knockdown of each gene was evaluated by real-time PCR (RT-qPCR) (see below for details) and presented in electronic supplementary material, figure S1.

### 4.3. *In situ* hybridization

For *in situ* hybridization, eggs were collected for 24 h and aged for additional 18 h to enrich for blastoderm and germband stage embryos (18–36 h after egg lay). Synchronized eggs were briefly washed with distilled water to remove debris and transferred to a 2 ml microtube with 1 ml of distilled water. The eggs were boiled for 90 s, the water was replaced by 1 ml of formaldehyde 12% (PBS 1X) and fixed for 2 h (6–8°C). After this period, the embryos were incubated with 1 ml of formaldehyde 4% containing 0,1% of Tween 20 under agitation for at least 1 h. Then the eggs are washed with PBST (PBS 1X, Tween 20 0,1%). After manual dechorionation with a fine forceps, embryos were stained with DAPI (1 µg µl^–1^), *in situ* hybridization was performed as in [65].

### 4.4. Total RNA extraction, cDNA synthesis and RT-qPCR assays

For cDNA generation, total RNA was extracted from 0 to 6, 18 to 24 or from 24 to 30 h embryos using Trizol Reagent (Invitrogen) as per manufacturer instructions. Total RNA was treated with RNAse free Turbo DNAse (Ambion, Life Technologies) to remove genomic DNA traces. cDNA was synthesized from 1ug total RNA using *in vitro* High-Capacity cDNA Reverse Transcription Kit (Applied biosystems). Quantitative Real Time PCR (RT-qPCR) was performed on a StepOnePlus Real Time PCR system (Applied Biosystems) using power SYBR-green PCR Master Mix (Applied Biosystems). The relative gene expression was calculated using the comparative *ΔΔ*CT method [66], using the ribosomal 18S (18S) and elongation factor 1 (Ef1) genes as reference genes as in [14]. The oligonucleotides used in RT-qPCR assays are listed in electronic supplementary material, table S1. All assays were conducted with biological triplicates and three to four technical replicates.

### 4.5. RNA-Seq analysis

Adult females were injected with double-stranded RNA for *Mal* (control) or Rp-*dpp* and blood fed the next day. After one week, adults were moved to new cages and embryos were collected for 6 h, comprising the stages between 0 and 6 h of development (cleavage stages). A subset of these embryos was fixed and DAPI stained to check for correct staging. Total RNA from two biological replicates was isolated with Trizol reagent (Invitrogen). RNA purification, reverse transcription, library construction and sequencing were performed at LACTAD-Unicamp facility (Campinas, Brazil) according to the manufacturer's instructions (Illumina, San Diego, CA, USA). RNA-Seq libraries samples were prepared using Illumina TruSeq v2 Kit (Illumina Inc., San Diego, CA, USA). Sequencing runs were performed in the Illumina HiSeq2500 sequencer platform (Illumina, Sand Diego, CA, USA) with 2 × 100 bp paired-end reads.

The RNA-Seq raw data were analysed with FastQC (https://www.bioinformatics.babraham.ac.uk/projects/fastqc/) considering sequence quality, read length, adapter content and k-mer content. Reads were cleaned from adapter sequences using Trimmomatic 0.39 [67] paired end mode adapter sequences from TruSeq RNA sample Prep kit v2 (Illumina). Paired end reads were trimmed with quality filters set to Q30 and minimum read length of 36 bp. Read mapping, transcript assembly and expression levels were performed using the workflows of HISAT2, StringTie and DESeq2 [68]. Hisat2 (version 2.2.1) [69] software was used for alignment of paired-end RNA-seq data to the kissing bug reference genome (Rhodnius_prolixus-3.0.3) [12] with *--dta* (downstream transcriptome assembly) option. All mapped samples were merged into one file for input to the StringTie [68] software for transcript detection based on existing gene annotation RproC version 3.5. Samtools (version 1.9) [70] was used for file format conversion between alignment and quantification steps.

The Python script prepDE.py (https://github.com/gpertea/stringtie/blob/master/prepDE.py) was used for read count extraction of each transcript from coverage values estimated by StringTie [68]. From prepDE.py results, differential expression analysis was performed using DESeq2 (version 1.38.2) [71] in the R environment (version 4.2.2). For identification of differentially expressed genes (DEGs), the criterion fold change ≥ 1.5 and *p*_adj_ ≤ 0.05 was adopted. Raw sequence reads were deposited at NCBI SRA as PRJNA924824 and the list of DEGs is available in the electronic supplementary material, table S2.

### 4.6. Image acquisition and processing

Microscopic images were obtained using a Leica MZ10F Stereomicroscope, on fixed embryos. Image treatment, analysis and overlay used Adobe Photoshop and ImageJ software.

### 4.7. Scanning electron microscopy

SEM was performed as in [72]. Briefly, unhatched eggs (greater than 15 days) were fixed by immersion in 2.5% glutaraldehyde (Grade I) and 4% freshly prepared formaldehyde in 0.1 M cacodylate buffer, pH 7.3. Samples were washed in cacodylate buffer, dehydrated in an ethanol series, and coated with a thin layer of gold. Models were observed in a Zeiss EVO 10 scanning electron microscope operating at 10 kV.

### 4.8. Statistical analysis

Experiments herein presented were performed at least three times unless stated otherwise. Phenotypes were quantified and results were analysed by One-way ANOVA for the comparison of two different conditions and One-way ANOVA followed by Tukey's test for the comparison among more than two conditions. Differences were considered significant at *p* < 0.05. All statistical analyses were performed using the Prism 7.0 software (GraphPad Software).

## Data Availability

Raw sequence reads were deposited at NCBI SRA as PRJNA924824. Additional information is provided in electronic supplementary material [73].
